# Rapid detection of *Mycobacterium ulcerans* with isothermal recombinase polymerase amplification assay

**DOI:** 10.1371/journal.pntd.0007155

**Published:** 2019-02-01

**Authors:** Michael Frimpong, Hubert Senanu Ahor, Ahmed Abd El Wahed, Bernadette Agbavor, Francisca Naana Sarpong, Kenneth Laing, Mark Wansbrough-Jones, Richard Odame Phillips

**Affiliations:** 1 Kumasi Centre for Collaborative Research in Tropical Medicine, Kwame Nkrumah University of Science and Technology, Kumasi, Ghana; 2 Division of Microbiology and Animal Hygiene, Georg-August University, Goettingen, Germany; 3 Institute for Infection and Immunity, St. George’s University of London, London, United Kingdom; 4 School of Medical Sciences, Kwame Nkrumah University of Science and Technology, Kumasi, Ghana; Stanford University, UNITED STATES

## Abstract

**Background:**

Access to an accurate diagnostic test for Buruli ulcer (BU) is a research priority according to the World Health Organization. Nucleic acid amplification of insertion sequence IS*2404* by polymerase chain reaction (PCR) is the most sensitive and specific method to detect *Mycobacterium ulcerans* (*M*. *ulcerans*), the causative agent of BU. However, PCR is not always available in endemic communities in Africa due to its cost and technological sophistication. Isothermal DNA amplification systems such as the recombinase polymerase amplification (RPA) have emerged as a molecular diagnostic tool with similar accuracy to PCR but having the advantage of amplifying a template DNA at a constant lower temperature in a shorter time. The aim of this study was to develop RPA for the detection of *M*. *ulcerans* and evaluate its use in Buruli ulcer disease.

**Methodology and principal findings:**

A specific fragment of IS*2404* of *M*. *ulcerans* was amplified within 15 minutes at a constant 42°C using RPA method. The detection limit was 45 copies of IS*2404* molecular DNA standard per reaction. The assay was highly specific as all 7 strains of *M*. *ulcerans* tested were detected, and no cross reactivity was observed to other mycobacteria or clinically relevant bacteria species. The clinical performance of the *M*. *ulcerans* (Mu-RPA) assay was evaluated using DNA extracted from fine needle aspirates or swabs taken from 67 patients in whom BU was suspected and 12 patients with clinically confirmed non-BU lesions. All results were compared to a highly sensitive real-time PCR. The clinical specificity of the Mu-RPA assay was 100% (95% CI, 84–100), whiles the sensitivity was 88% (95% CI, 77–95).

**Conclusion:**

The Mu-RPA assay represents an alternative to PCR, especially in areas with limited infrastructure.

## Author summary

Current diagnostic methods to detect *M*. *ulcerans* suffer from delayed time-to-results in most endemic countries by the prolonged period of time for the shipment and storage of samples to a distant, centralized laboratory. The *M*. *ulcerans* recombinase polymerase amplification assay (Mu-RPA) is a new, rapid diagnostic test developed for the detection of *M*. *ulcerans* infection, known commonly as Buruli ulcer, a chronic, debilitating, necrotizing disease of the skin and soft tissues. This assay is suitable for use on a portable detection device, with the potential to be used for quick diagnosis at the point of need, providing timely results to health workers at Buruli ulcer treatment clinics.

## Introduction

Buruli ulcer (BU) is a neglected tropical disease caused by *M*. *ulcerans*. The pathogenesis of BU is linked to the production of a polyketide toxin known as mycolactone which is cytotoxic and has immunomodulatory properties [1]. The disease affects mostly children and adults of all ages and presents as nodules, plaques, ulcers and oedema. There is a wide differential diagnosis ranging from lipomas, ganglion, onchocerciasis nodules, and fungal lesions for non-ulcerated lesions to tropical, diabetic or vascular ulcers in the case of ulcerated lesions so it is vital that accurate diagnosis should be available close to patients in rural West Africa [2,3].

Currently, there are no preventive strategies against BU as the mode of transmission remains unknown and there is no vaccine. However, antimicrobial therapy with a combination of rifampicin and streptomycin or clarithromycin has proven effective in healing all forms of the disease and reducing the recurrence rate to less than 2% [4–6]. As a result, early diagnosis of clinically suspected cases has become a critical step in the clinical management of BU in order to prevent misdiagnosis and administration of unnecessary antibiotics [7].

The gold standard diagnostic tool for BU is PCR for the repeat sequence IS*2404*, which is specific to *M*. *ulcerans* [8]. Microscopy for acid fast bacilli and culture for *M*. *ulcerans* have low sensitivity and histopathology is rarely available in endemic areas. Although PCR has high sensitivity (up to 95%) it has to be performed in a reference laboratory, often far from the endemic area, due to the need for a sophisticated laboratory setup and skilled personnel which may not be available in endemic communities [9]. Difficulties with sample collection and transportation may lead to a slow turnaround resulting in delayed treatment and an increase in costs. A field friendly diagnostic tool would bring diagnosis closer to the patients, thereby reducing costs and bringing forward the start of treatment.

Recently, isothermal amplification techniques such as loop-mediated amplification (LAMP) have been proposed as an alternative to PCR for diagnosis of BU [9,10]. Unlike PCR, isothermal amplification techniques do not require a thermocycler and yield readily readable results within a short turnaround time. Recombinase polymerase amplification (RPA) has emerged as a novel isothermal technique in molecular diagnosis of various infectious diseases [11] including *tuberculosis* [12,13] and *paratuberculosis* (Johne’s disease) [14]. Compared to PCR and other isothermal techniques RPA is more rapid (less than 20 min) and simpler to run as it requires a lower temperature (37–42°C) [11]. This technique opens the door to extending molecular diagnosis in fieldwork and at the point of care.

In this study, we developed a real-time isothermal RPA assay for the detection of *M*. *ulcerans* as an alternative to PCR. The efficiency of the assay as a diagnostic tool was determined by testing its sensitivity and specificity with clinical samples from suspected patients.

## Materials and methods

### Preparation of molecular standard

To generate a molecular standard that will be used as positive control and a calibration template with known copy numbers, a 451 bp fragment of IS*2404* sequence covering the nucleotides 96540 to 96990 (Genbank accession no. CP000325.1) was synthesised by GeneArt Gene Synthesis (Invitrogen, Regensburg, Germany). A dilution range of 10^0^ to 10^6^ copies/μl of the standard was prepared.

### DNA extraction from bacterial strains and clinical samples

Genomic DNA was derived from mycobacterial strains obtained from Belgian Co-Ordinated Collections of Micro-organisms, Institute of Tropical Medicine (BCCM/ITM Mycobacteria Collection, Antwerp, Belgium). DNA was extracted from pure colonies following culture on Lowenstein-Jensen slant Medium (BD, Franklin Lakes, NJ, USA) for 4–6 weeks. Colonies were suspended in 700 μl of Cell Lysis solution (CLS) (Qiagen, Hilden, Germany) and stored in a fridge until ready for extraction. Similarly, swabs and fine-needle aspirates obtained from ulcerative and non-ulcerative lesions respectively were collected directly into 700 μl CLS. *M*. *ulcerans* DNA was extracted using the Gentra Puregene DNA isolation kit (Qiagen, Hilden, Germany) following manufacturer’s instructions with minor modifications as previously described [15]. The QIAamp DNA Mini Kit (Qiagen) was used for isolation of DNA from 5 bacterial species frequently colonizing human skin following manufacturer’s instruction. The amount of DNA was measured with a DeNovix DS-11 Spectrophotometer (DeNovix Inc., Wilmington, USA).

### Real-time PCR

Real-time PCR was run on a Rotor-Gene Q (Qiagen, Hilden, Germany) using the Hot FIREPol Probe qPCR Mix Plus to amplify a 59 bp long fragment (Nucleotide 96627 to 96685 of the GenBank accession number CP000325.1) (see Table 1). The PCR reaction volume was 20 μl containing 1 μl each of 10 μM IS*2404* TF and IS*2404* TR, 1 μl of 5 μM IS*2404* TP2, 4 μl of 5 U/μl qPCR Mix Plus, 2 μl of 10x Exo IPC Mix, 0.4 μl Exo IPC DNA and 8.6 μl Molecular grade H_2_O as well as 2 μl of the DNA template. The PCR cycling conditions were adopted from a published assay [16] as follows: Initial denaturation for 15 minutes at 95°C, then 40 cycles of 95°C for 15 seconds and 60°C for 60 seconds with fluorescence activation. Each batch of samples run included negative extraction control, no template control (NTC) and positive control. All the negative extraction controls and NTC did not show an amplification curve. This indicated that there were no contamination during preparation of the PCR master mixes or during the DNA extraction process. All samples that did not show an amplification curve above the set threshold were considered negative. Similarly, the internal positive control (IPC) and the positive controls showed exponential amplification curves. All samples with an exponential amplification curve with the cycle threshold (CT) less than 40, was considered positive.

**Table 1:** Primers and probes for the RPA and real-time PCR for amplification and detection of *Mycobacterium ulcerans*.

Primer/Probe	Sequence (5'-3')	Gene target	Nucleotide position	Amplicon size	Reference
IS*2404*TF	*AAA GCA CCA CGC AGC ATC T*	IS*2404*	96685–96667		[16]
IS*2404*TR	*AGC GAC CCC AGT GGA TTG*		96627–96644	59 bp	
IS*2404*TP	*FAM-CCG TCC AAC GCG ATC GGC A-BBQ*		96664–96646		
Mu_RPA F1	*ATG CAT CGC ATC CAC AGT GAC CAG CCA CCG*	IS*2404*	96857–96828		This study
Mu_RPA R2	*ATT GGT GCC GAT CGC GTT GGA CGG CAA GAT G*		96641–96671	217 bp	
Mu_RPA P	*GTA GGC GAA CAC CGA CAC GAG ATG CGT GGC QTF CGC TTT GGC GCG TA–PH*		96731–96685		

QTF are sites of the quencher and fluorophore in the order quencher BHQ1-dt (Q), THF (T) and Fam-dT (F)

FAM, Carboxyfluorescein fluorescent dye; BBQ, BlackBerry Quencher; dT-BHQ1, Black Hole Quencher-1; dT- deoxythymidine; THF, Tetrahydrofuran

### RPA assay design and conditions

The Mu-RPA assay was designed to target the insertion sequence IS*2404* which has been shown to have high sensitivity and specificity for diagnosing BU. Primers were designed using the recommendations given in the TwistDx instruction manual [17] and Primer-BLAST available at http://www.ebi.ac.uk/ena/data/sequence/search combining Primer3 and BLAST global alignment. Oligonucleotide primers and probes were synthesized by TIB MOLBIOL (Berlin, Germany). Preliminary screening of 3 forward primers and 3 reverse primer combinations were tested with the Twist Amp Basic “Improved Formulation” kits according to the manufacturer’s instructions (TwistDx Ltd., UK) in a final reaction volume of 50 μl. Briefly, 29.5 μl Rehydration buffer, 8.2 μl H_2_O, 2.4 μl of 5 μM of both forward and reverse primer and 5 μl of the DNA template were added to a freeze dried reaction pellet. Water was used for the negative control. The RPA reactions were incubated at 42 °C for 15 minutes following the addition of 2.5 μl 280 mM MgAc. The template used was 1 ng/μL of *Mu* strain (ITM 063846). One primer pair producing a 217 bp fragment of gene IS*2404* sequence covering nucleotides 96641–96857 (Genbank accession CP000325.1) was selected (Table 1) after analysis of amplicons by agarose gel electrophoresis (AGE).

In the case of real-time RPA detection, TwistAmp Exo “Improved Formulation” kit (TwistDX Ltd, Cambridge, UK) was used according to the protocol described dx.doi.org/10.17504/protocols.io.vvve666. Fluorescence detection at 570 nm for FAM channel was measured and a threshold set by increasing the fluorescence above the 3 standard deviations over the background detected in the first minute of incubation. We programmed the T8- fluorometer using the T8-ISO Desktop application (Axxin Pty Ltd, Victoria, Australia) to detect the lowest dilutions that met criteria for distinguishing positive samples from negative controls based on serial dilutions of the molecular standard. All tests were run at 42 °C for 900 seconds (15 minutes) with mixing after 4 minutes. To be considered positive, a sample required either an amplitude or gradient of at least 900 mV over a 40 second sliding window during the amplification phase of the test (300–900 seconds).

### Analytical sensitivity and specificity

To determine the analytical sensitivity, 5 μl and 2 μl of a dilution range 10^6^–10^0^ copies/μl of quantitative IS*2404* DNA fragment standard was tested six times in triplicates with Mu-RPA and the real-time PCR, respectively. The threshold time (TT) and the cycle threshold (CT) values were plotted against the number of molecules detected. Non-regression analysis and probit analysis was done by GraphPad Prism (GraphPad software, San Diego, USA). The limit of detection in 95% of the dilutions was extrapolated from the sigmoid curve. To assess the specificity of the Mu-RPA assay for the detection of *M*. *ulcerans*, 1 ng/μL DNA from closely related mycobacterial species and bacterial species contaminating the human skin were tested.

### Assessment of RPA performance using clinical samples

RPA was evaluated with DNA extracts isolated from samples previously obtained from patients referred to BU treatment centres in Ghana. Samples were collected as part of routine diagnostic procedure. Swabs were obtained from ulcers and fine needle aspirates from non-ulcerative lesions using standard guidelines [18]. In total, a panel of 79 DNA extracts from 69 clinically suspected BU patients and 12 clinically confirmed non BU ulcers. All samples were tested with both the real-time PCR and the Mu-RPA assay to determine the clinical sensitivity and specificity of the assay using real-time PCR as the reference test. Both tests were conducted independently by two different investigators and were blind to the results of the other test.

### Statistics

Microsoft Excel 2016 was used for data management. GraphPad Prism v.6 (GraphPad software, San Diego, USA) was used to calculate a semi-log regression of the dataset of repeated amplification runs of RPA and real-time PCR by plotting the mean threshold time (TT) and cycle threshold (CT) respectively against molecules detected of the standard DNA dilutions (10^6^−10^0^ copies/μl). A probit regression analysis was performed to determine the limit of detection (LOD) in 95% of dilutions for both assays using GraphPad Prism. Descriptive statistics were used to obtain general descriptive information such as median and interquartile ranges from the data. Contingency tables and receiver operating characteristics (ROC) curve analysis were employed to calculate the sensitivity, specificity and the predictive values in evaluating the assay.

### Ethics statement

Ethical approval for this study was obtained from the Committee on Human Research, Publication and Ethics (CHRPE/AP/122/17) School of Medical Sciences, Kwame Nkrumah University of Science and Technology. A total of 79 samples were included in this study under ethical consideration. All samples were handled anonymously.

### Accession number

*Mycobacterium ulcerans* Agy99, Complete genome–Genbank accession no. CP000325.1.

## Results

### RPA assay for *M*. *ulcerans* detection

Primers and probes were designed to target the insertion sequence, IS*2404*, a region that has been shown to have high sensitivity for diagnosing BU using PCR. The region chosen for the RPA primer design was overlapping with already published primer binding sites of *M*. *ulcerans* PCR [19]. Designed primers and probes were screened using BLASTN and the NCBI nucleotide database to ensure that target sequences for the designs were exclusive for *M*. *ulcerans* strains. The RPA assay was developed in single tube reactions to screen primers and test different reaction conditions. Among 3 forward primers, 3 reverse primers and one probe (P) only F1+R2 and probe (Table 1) were able to amplify down to 10 DNA molecules per reaction. The target region was subjected to a BLAST search (blast.ncbi.nlm.nih.gov) to check for cross-reactivity with non-*Mu* sequences and no non-*Mu* sequences were identified.

**Fig 1:**
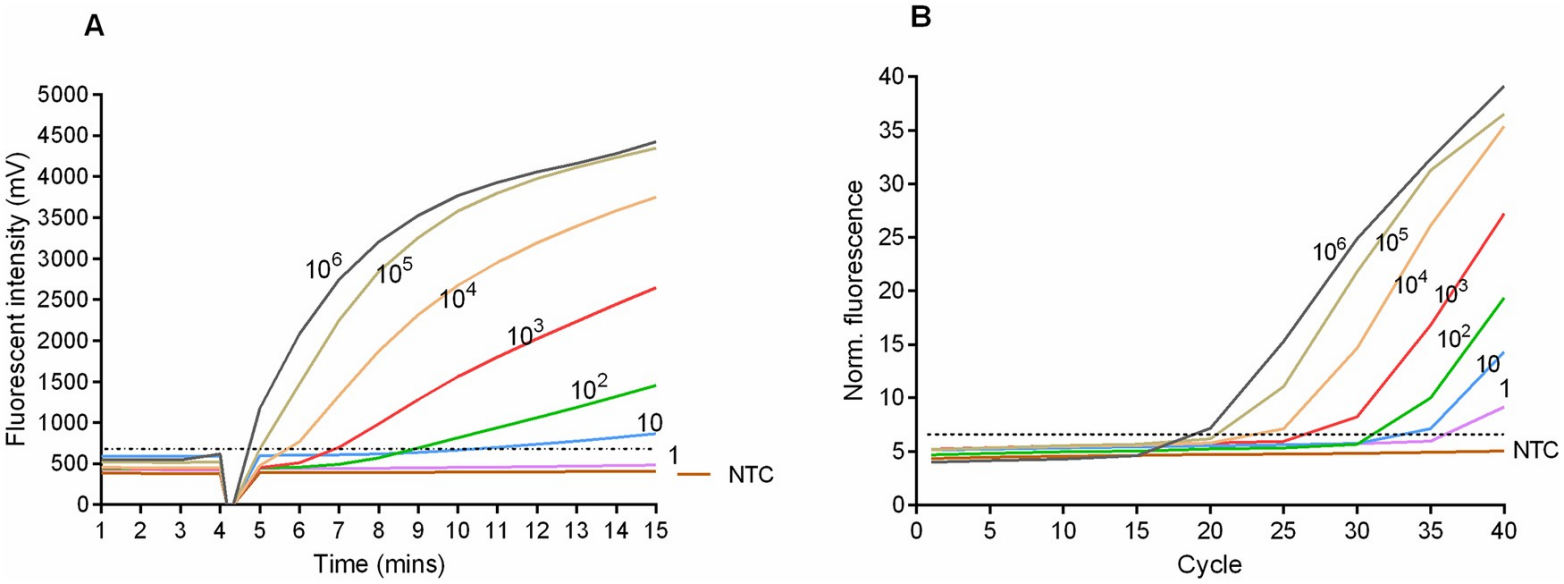
Amplification curves in Mu RPA (A) and BU real-time PCR (B) assays applying serial dilutions (10^6^–1) of the molecular DNA standard. No fluorescent signals were detected in RPA before four minutes because the strip was taken out for mixing. The lowest number of copies detected for RPA and real-time PCR was 10 and 1 respectively.

**Fig 2:**
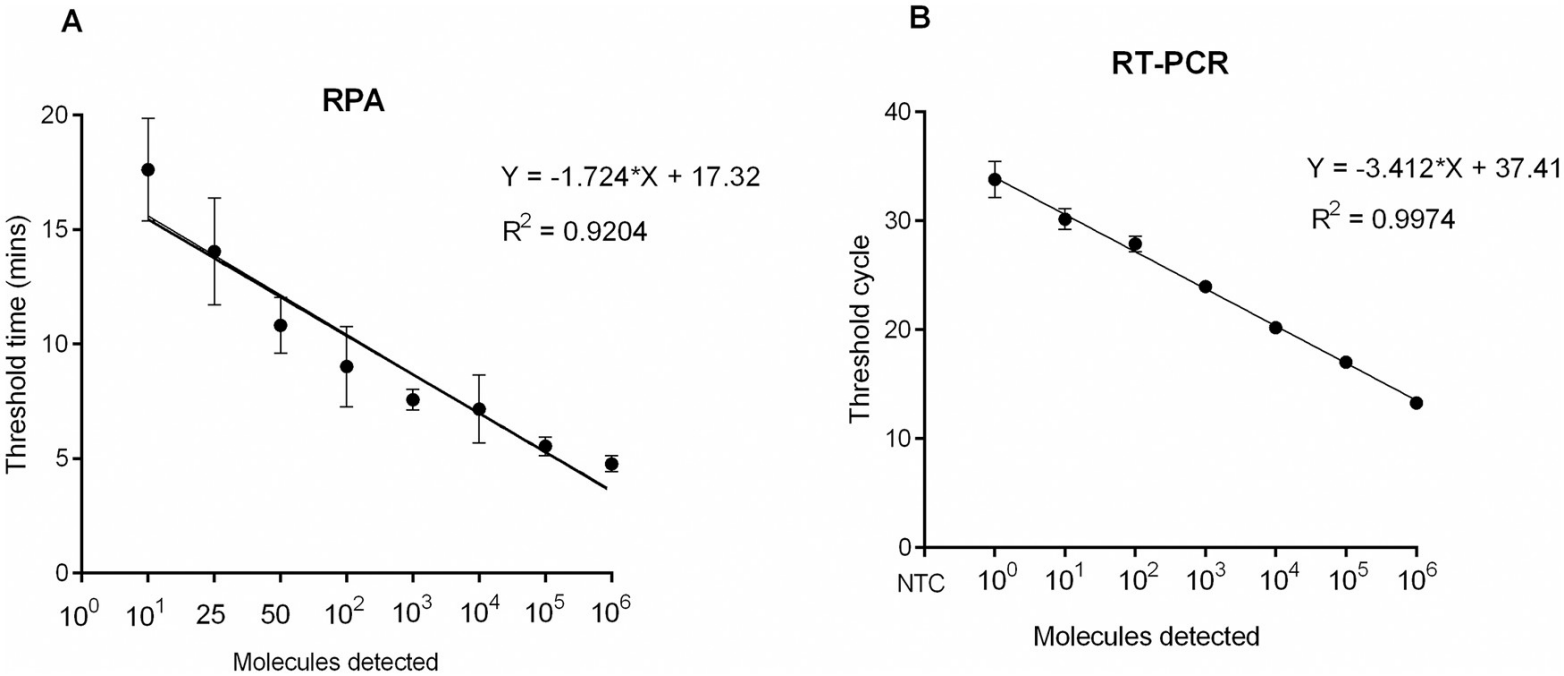
Reproducibility of the RPA and real-time PCR assays using data set of 6 runs of serial dilution of the MU molecular standard. A: Threshold time (Y-axis) was plotted versus the log_10_ concentration of molecules (X-axis) for the RPA. B: Threshold cycle (CT) on the Y-axis was plotted versus the log_10_ concentration of molecules (X-axis) for the real-time PCR. RPA assay produced results between 5 to 20 minutes. The error bars represent the range. The real-time PCR had a higher efficiency compared to the RPA, due to the regular cycle format of the PCR, while there is no strict separation of the amplification cycles in the isothermal RPA technology.

**Fig 3:**
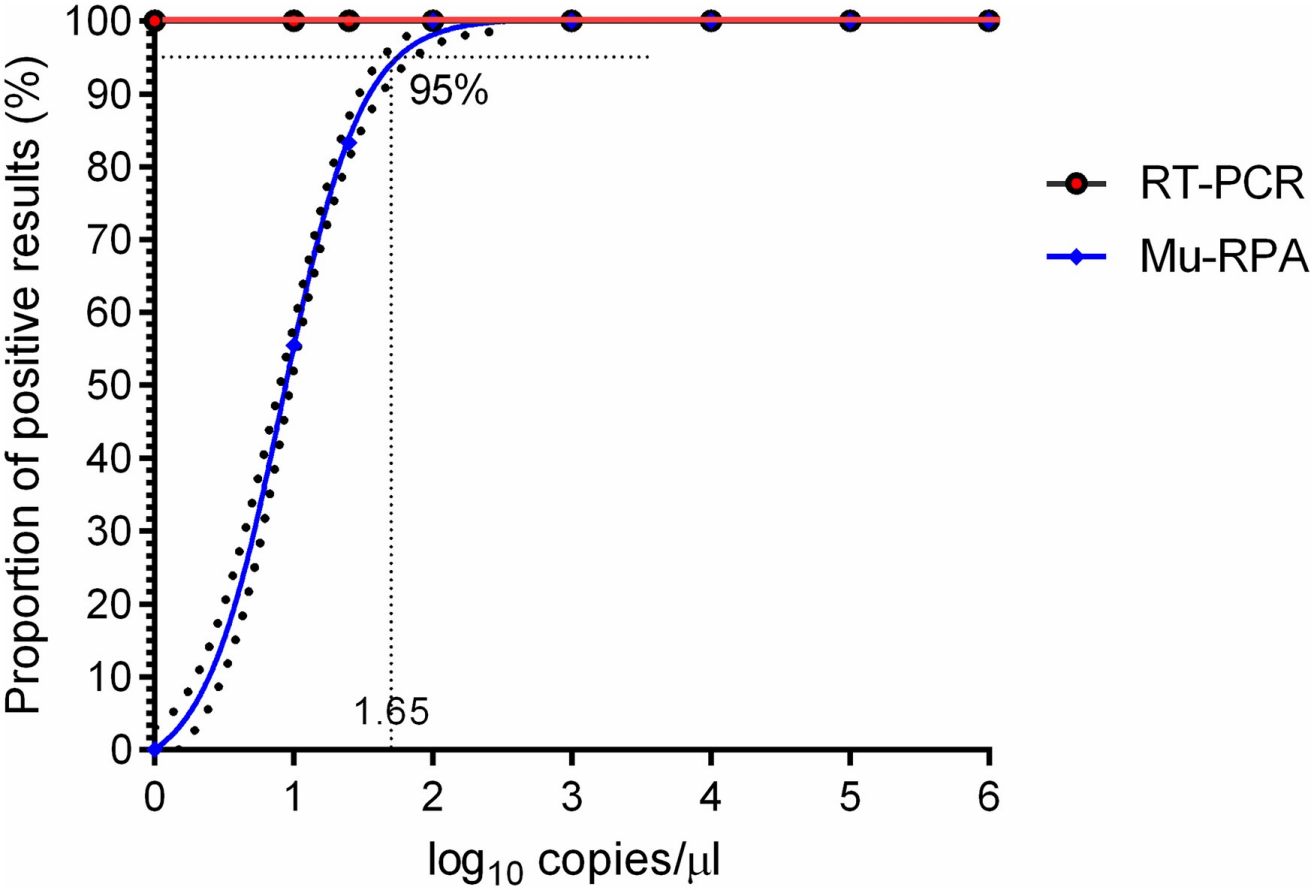
Probit curve used to calculate the limit of detection of the Mu RPA assay. IS2404 DNA fragment standard was tested in dilution range 10^6^–10^0^ copies/μl of six times for each dilution. Percent detection (Y) was plotted versus log_10_ concentration. The limit of detection of RPA at 95% of dilutions is 45 copies/μl was extrapolated from the sigmoid curve.

The detection limit of the Mu-RPA assay was determined by six independent runs of serial dilutions (106–10^0^ copies/μl) with the molecular standard (Fig 1). Rapid detection was observed; 5 minutes for the detection of 10^6^ copies, 7 minutes for 10^3^ copies and 12 minutes for 10 copies. The data were used in semi-regression and probit regression analyses. The assay was reproducible showing good correlation between copy number and time to detection (Fig 2) but with less efficiency compared to PCR (R^2^ 0.92 versus 0.99 respectively). The limit of detection in 95% of dilutions was 45 copies (Fig 3).

To determine whether there was any cross-reactivity of the Mu-RPA assay for other microorganisms other than *M*. *ulcerans*, DNA extracts from a panel of 7 non-*M*. *ulcerans* mycobacterial species, 5 bacterial species that commonly colonise the skin and 7 *M*. *ulcerans* strains were tested. No amplification signals were obtained from any of the bacterial DNAs other than those of the *Mu* strains (Table 2).

**Table 2:** Bacteria species and strains used for testing cross reactivity of the Mu-RPA assay.

Name of bacteria	ITM No.	Origin	RPA results
*Mycobacterium ulcerans*	063846	Benin	+
*Mycobacterium ulcerans*	C05150	DRC	+
*Mycobacterium ulcerans*	C05142	Australia	+
*Mycobacterium ulcerans*	C08756	Japan	+
*Mycobacterium ulcerans*	092078	French Guiana	+
*Mycobacterium ulcerans*	070290	China	+
*Mycobacterium ulcerans*	083720	Mexico	+
*Mycobacterium marinum*	12562		−
*Mycobacterium tuberculosis*	000092		−
*Mycobacterium avium*	960261		−
*Mycobacterium vaccae*	M001002		−
*Mycobacterium africanum*	M002046		−
*Mycobacterium chelonae subsp*. *Complex*	940717		−
*Mycobacterium celatum*	Clinical strain	UK	−
*Staphylococcus aureus*	ATCC 25923*		−
*Escherichia coli*	ATCC 25922*		−
*Pseudomona aeruginosa*	ATCC 27853*		−
*Proteus vulgaris*	ATCC 13315*		−
*Staphylococcus epidermidis*	ATCC 1228*		−

Results of Mu RPA of DNA extracts; “+” indicates a positive and “-” indicates a negative test results

*—ATCC bacterial strains obtained from the bacteriology unit of KCCR.

### Analysis of diagnostic accuracy

The assay performance as a diagnostic tool was assessed using a panel of 67 DNA samples collected from suspected cases of BU during routine care by an expert and 12 non-BU lesions clinically confirmed to have other diseases (ie. 3 diabetic foot ulcers, 4 traumatic injuries, 3 cellulitis and 2 surgical site infections). The samples were obtained from patients presenting with varied lesions: 12 (15%) nodules, 9 (11%) plaques, 1 (2%) edema and 57 (72%) ulcers. It was made up of 24 (30%) fine needle aspirates (FNA) and 55 (70%) swabs. The characteristics of cases and the type of samples collected are summarized in Table 3.

**Table 3:** Demographic and clinical characteristics of patients whose samples were used in the study.

	No. (%) of total lesions (n = 79)
**Sex**	
Male	38 (48)
Female	41 (52)
**Sample type**	
FNA	24 (30)
Swab	55 (70)
**Age in years**	
Median (IQR)	17 (10–42)
**Type of Lesion**	
Nodule	12 (15)
Plaque	9 (11)
Edema	1 (2)
Ulcer	57 (72)
**WHO Category of Lesion**	
I	32 (40)
II	29 (37)
III	18 (23)

IQR, Interquartile range; I, A single lesions ≤ 5 cm in diameter; II, A single lesion 5–15 cm in diameter; III, A single lesion >15 cm in diameter, multiple lesions, critical sites, osteomyelitis

**Fig 4:**
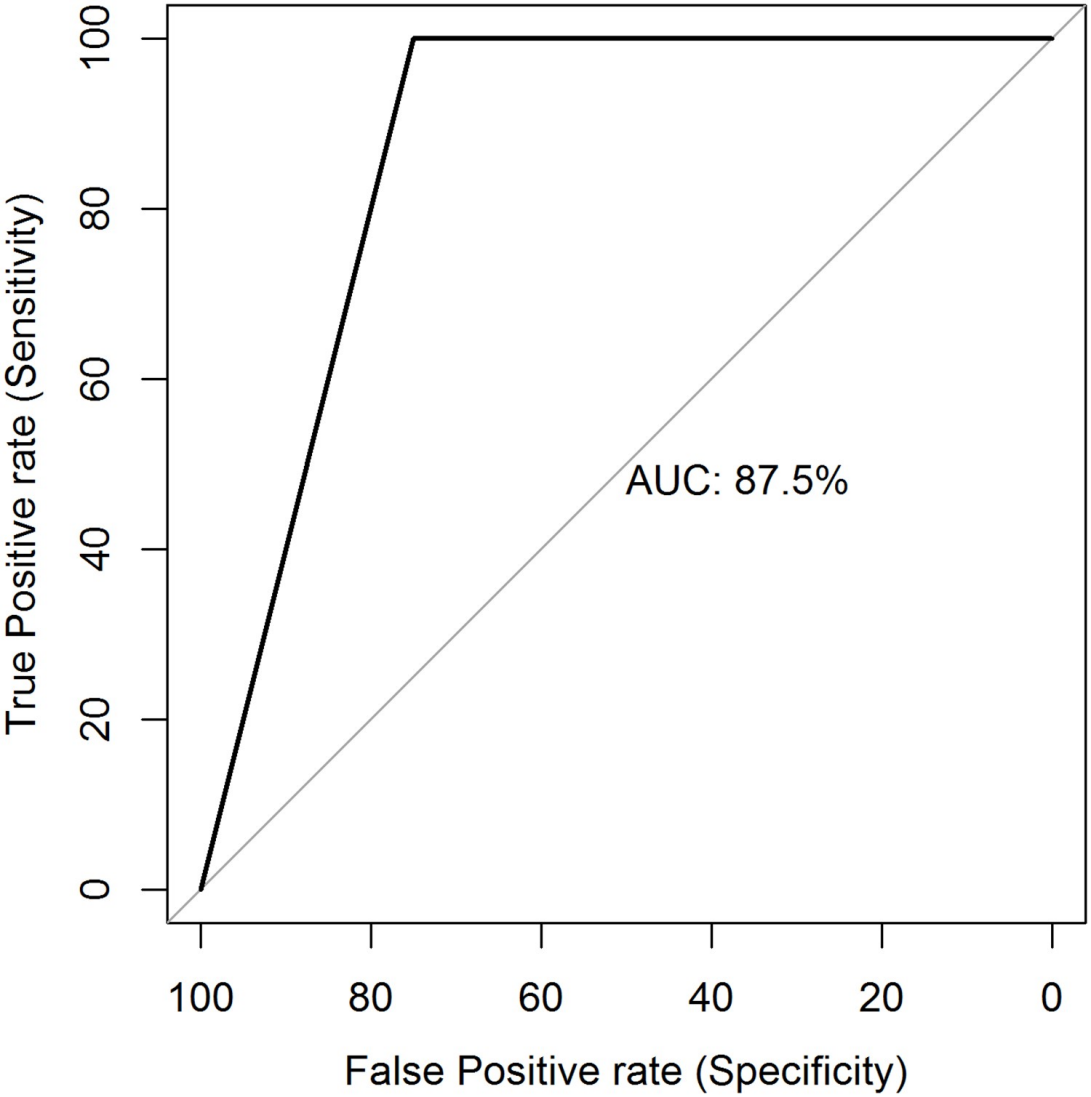
Receiver operating characteristic curve for the Mu-RPA assay. The x-axis shows the values for specificity and the y axis, the values for sensitivity. The AUC was 88 (95% CI 70–96).

All samples were tested by both RPA and real-time PCR. Fifty-eight of these samples were confirmed by PCR as BU. Of the 58 confirmed cases, 51 were correctly identified by the RPA assay with 7 false negative results giving a sensitivity of 88% (95% CI, 77–95). The 21 PCR negative samples were all negative by RPA, specificity of 100% (95% CI, 84–100) and a 100% (95% CI, 93–100) positive predictive value (PPV) with a Youden’s index of 88% (95% CI, 61–95). When the analysis was stratified by type of sample, the sensitivity and specificity of the RPA for swabs in comparison to PCR were 92% (95% CI 78–98) and 100% (95% CI, 82–100) respectively with a 100% (95% CI, 89–100) PPV. Similarly, the sensitivity and specificity of FNA samples were 82% and 100% (95% CI, 81–100) respectively (See Table 4). The specificity was 100% for all forms and severity of lesions analyzed. To further assess the reliability of the assay, we plotted the receiver operating characteristic (ROC) curve (Fig 4). The area under the curve (AUC) was 88 (95% CI, 70–96).

**Table 4:** Clinical sensitivity and specificity of Mu-RPA compared to real-time PCR for IS*2404* (stratified by type of sample used).

	PCR +ve	PCR–ve	Total	Sensitivity % (95% CI)	Specificity % (95% CI)	PPV % (95% CI)	NPV % (95% CI)
**Swab**							
Mu RPA +ve	33	0	33	92 (78–98)	100 (82–100)	100 (89–100)	86 (65–97)
Mu RPA–ve	3	19	22				
**FNA**							
Mu RPA +ve	18	0	18	82 (60–95)	100 (16–100)	100 (81–100)	33 (0.4–78)
Mu RPA–ve	4	2	6				
**Total**							
Mu RPA +ve	51	0	51	88 (77–95)	100 (84–100)	100 (93–100)	75 (55–89)
Mu RPA–ve	7	21	28				

PPV, Positive predictive value; NPV, Negative predictive value; +ve, Positive; -ve, Negative

## Discussion

A major control strategy for BU is early detection and treatment, hinging on effective laboratory confirmation of suspected cases, since prevention is not possible in the absence of either an effective vaccine or a clear understanding of the mode of transmission. New diagnostic tools for confirmation of cases that can be implemented at the district health facility level where most patients are treated is a priority for the WHO. Since its discovery in 2006 by Piepenburg and his colleagues [20], RPA has been explored for the molecular diagnosis of infectious diseases caused by pathogenic bacteria such as methicillin-resistant *Staphylococcus aureus*, group B streptococci, *Francisella tulerensis*, *Yersinia pestis*, *Bacillus anthracis*, Pse*udomonas aeruginosa* [21,22], C*hlamydia trachomatis*, *Mycobacterium tuberculosis*, *Mycobacterium avium* subsp. *paratuberculosis* [12–14] and parasitic infections caused by *Plasmodium falciparum*, *Entamoeba histolytica*, *Leishmania donovani* and *Toxoplasma gondii* and *Schistosoma japonicum* [23–28].

In this paper we have described the development of a rapid RPA test targeting the IS*2404* gene of *M*. *ulcerans* that successfully detects all strains analyzed. The highest burden of Buruli ulcer disease is in West Africa, but it is a global disease that has been reported in about 33 countries in Africa, South America and the Western Pacific regions including Asia and Australia, with about 15 countries reporting cases annually [29]. The ability of the RPA to detect all strains from different parts of the globe makes it an optimal performance feature of a new diagnostic test for BU. The limit of detection using a molecular standard was 45 target copies. While the analytical sensitivity is lower than that of real-time PCR (1–10 copies), we believe it is still within the clinical detection range of most symptomatic BU patients, as demonstrated by the high (88%) clinical sensitivity and specificity (100%) using clinical samples. The sensitivity is higher than previously reported for laboratory tests currently available for use at the district level, such as 40–60% [30,31] for smear microscopy detecting acid-fast bacilli and 73% [32] for fluorescent thin-layer chromatography detecting mycolactone.

Several studies have demonstrated that bacterial super infections occur in Buruli ulcer lesions. The most common bacteria isolates include *Staphylococcus aureus*, *Pseudomonas aeruginosa* and *Klebsiella pneumoniae* [33–35]. When Mu-RPA was tested against a range of skin contaminants including these, it showed high specificity for *M*. *ulcerans*, as well as failing to react with any of the closely related mycobacteria tested. In this respect it was similar to the PCR for IS*2404* currently regarded as the gold standard for confirmation of BU disease.

A similar point of care method known as the Loop mediated isothermal amplification method (LAMP) has comparable sensitivity to Mu-RPA but with a turnaround time of over 60 minutes for DNA amplification alone [9,10]. The assay employs six primers to amplify the IS*2404* gene at a high temperature of (60 °C). The results readout relies on changes in assay turbidity, which is recognized by the naked eye or recorded in real-time by a portable device. In contrast, the use of two opposing primers and a probe in the Mu-RPA allows exponential amplification of target sequence with high efficiency similar to that of real-time PCR but it is faster and more portable. The RPA can also be multiplexed for detection of other pathogens or drug resistance profiles if required, a feature that is lacking with LAMP. Due to its simplicity and the use of less energy intensive equipment suited for low resource setting, RPA has been proposed as an alternative to PCR for point of care diagnosis of infectious diseases [24,36]. Like other isothermal techniques, RPA tolerates PCR inhibitors present in less purified samples [28,37], so it can be used with crude sample preparation techniques to facilitate diagnosis in low resourced settings. This has not been assessed in the present study. Further studies will be needed to fully evaluate the possible use of this assay with crude material, including careful consideration of biosafety, sensitivity and DNA contamination. There is also a need to examine simpler methods for disrupting mycobacterial cells to allow access to bacterial nucleic acid either with reagent, small device or both.

### Conclusion

A real-time RPA assay was developed for the rapid and accurate detection of *M*. *ulcerans* DNA with high sensitivity, specificity and reproducibility comparable to real-time PCR. It was significantly faster than available real-time PCR methods for detecting *M*. *ulcerans* with a run time of 15 minutes, compared to almost 2 hours for real-time PCR. Potentially the Mu-RPA can be used in a low resource setting closer to the patients when combined with a fast DNA extraction method.

## Supporting information

S1 File IS*2404* nucleotide 96108–97471 reverse strand with position of Mu-RPA primers and probe highlighted. (PDF)

S2 File STARD checklist. (DOCX)

S3 File Flow diagram of participants included in the validation of the index test. (PPTX)

S4 File Raw data of participants included in the study. (XLSX)
