# Supplementary material for: Rapid detection of Mycobacterium ulcerans with isothermal recombinase polymerase amplification assay
Source: PLoS Negl Trop Dis. 2019 Feb 1;13(2):e0007155. doi: 10.1371/journal.pntd.0007155 (PMC6373974; doi:10.1371/journal.pntd.0007155)
Supplement: S1 File — (PDF) [file pntd.0007155.s001.pdf]

IS*2404* nucleotide 96108-97471 reverse strand

1 cagggctccg gcgttgttga ttagcaggct tgtgagctgg ggaattgtgg attgaggagg

61 tctaggcggc ggttggcggt caaagcggtg atccggcagg cgtcggcgat gttgtcggcg

121 ccgttgaggc ggtgcagatt gatcgcggtg ttgcgtagcg ttgctaggac ctgtgcgccg

181 tttccggtat gtgccctgtg acggtcttcg tcgaaggtga cgtcgcgtat ccagtgcagg

241 ctgttctcga ttccccagtg ttgacgcatc caggtcatga tcgcggtagg gcgggcgtgc

301 tcgaacggca ggctgcagat ggcatagacc acctccacgc tgcgctggtc ggtggcggtg

361 atcaagcgtt cacgagtgat ccggatgatt tgttttgcgt aggggaagcc gattcctcgt

421 gcagcggtga tgatttgcag ggtgcgggtc tcgacacggc cgtggccgcg ggagtcgtcg

481 gtagcggctg cgggcacctc ggcccagggc agcgcggtga tacgggcaag tattttggct

541 tggttggact tgacgatcat caggtagtgc gacttcaagg tggcgcagat caacttcgcg

601 gtgacgacct gggt**atgcat cgcatccaca gtgaccagcc accg**caagct acccggcagc

661 agcgtgagca gggcacgtac gcagggaatt tcattgcttt tctcggcgac agcgagttga

721 ccgagcacca atcgggcacg **gtaggcgaac accgacacga gatgcgtggc tgtcgctttg**

781 **gcgcgta**aag caccacgcag ***catctt*****gccg tccaacgcga tcggcaccaa t**ccactgggg

841 tcgctgctgg ccacgtgtgc agtgaagtaa ctgcccatcc tggcgttgag gtcggcgggg

901 tctagccgag acaaaacagc gcggaaggtc ttctcactgg gccgccggaa ccggacccct

961 aattgggcca acacatcatc ggaagcggtg gccgcccatg tggcaaaacc agcatagccg

1021 cgcatccccg cggcagtggc cagaaccgcg atcgccaaca aagccatcag cgaatacctc

1081 cgaccgcgtg ggtccctcgg gtctggcacc gaggccaaca catcgagaaa ctccgatccg

1141 atgagctcgc tgcgctcacc agcactccag cggtcgaaca cctctggaac cgacgacaca

1201 ggcaatactg aaacgctagg cacgggcaac tccagaccaa cgacgaacag taaccaactc

1261 gatcgttctc cagatagccc gtgcctacac tccggcccca atagcgcggc accaaagatc

1321 atcaacccag ctcacaagcc tgctaatcaa caacgccgga gccc
