# Supplementary material for: Rapid detection of Mycobacterium ulcerans with isothermal recombinase polymerase amplification assay
Source: PLoS Negl Trop Dis. 2019 Feb 1;13(2):e0007155. doi: 10.1371/journal.pntd.0007155 (PMC6373974; doi:10.1371/journal.pntd.0007155)
Supplement: S3 File — (PPTX) [file pntd.0007155.s003.pptx]

## Slide 1
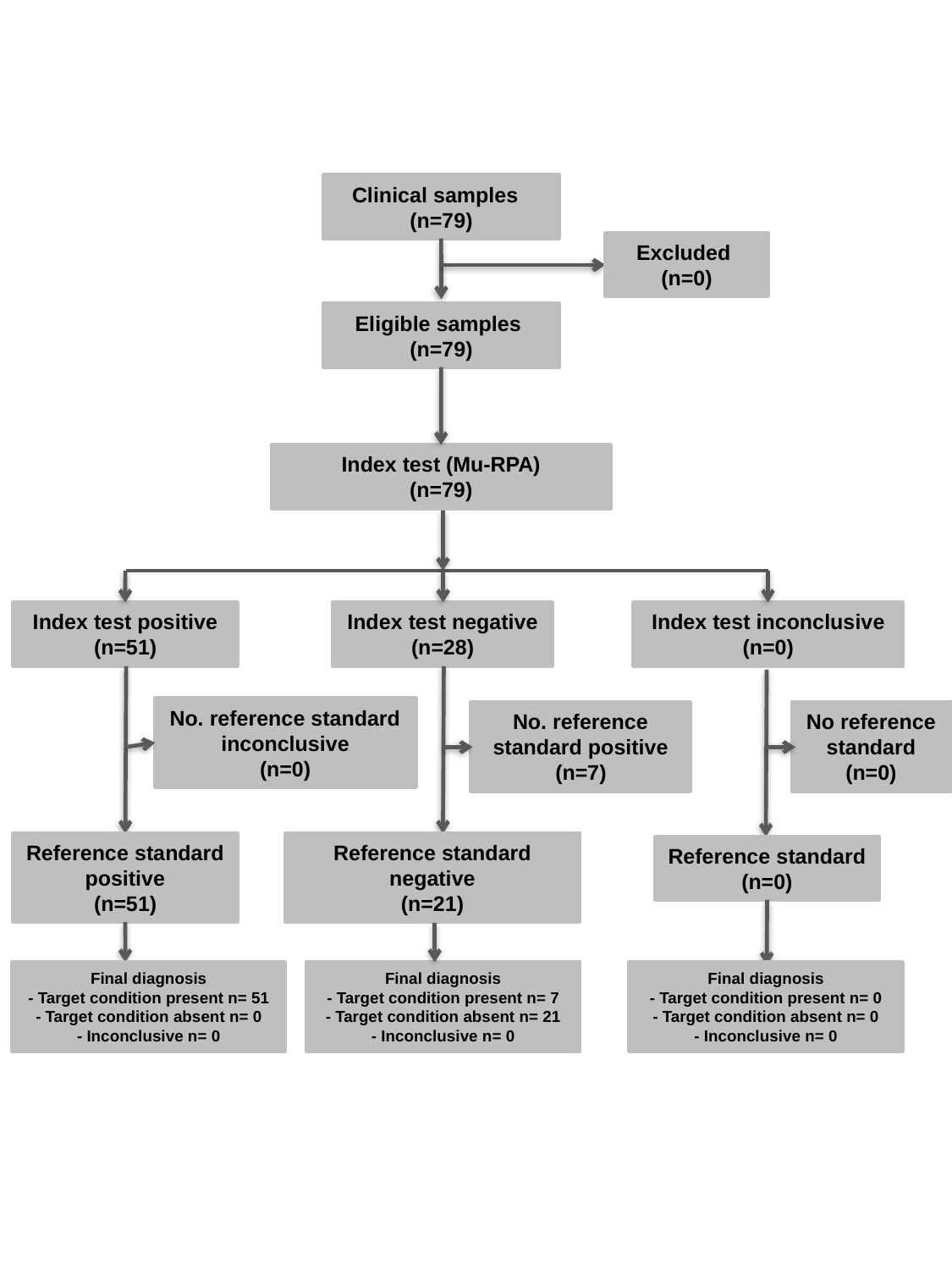

Clinical samples
(n=79)
Excluded
(n=0)
Eligible samples
(n=79)
Index test (Mu-RPA)
(n=79)
Index test positive
(n=51)
Index test negative
(n=28)
Index test inconclusive
(n=0)
No. reference standard inconclusive
(n=0)
No. reference standard positive
(n=7)
No reference standard
(n=0)
Reference standard positive
(n=51)
Reference standard negative
(n=21)
Reference standard
(n=0)
Final diagnosis
- Target condition present n= 51
- Target condition absent n= 0
- Inconclusive n= 0
Final diagnosis
- Target condition present n= 7
- Target condition absent n= 21
- Inconclusive n= 0
Final diagnosis
- Target condition present n= 0
- Target condition absent n= 0
- Inconclusive n= 0
